# Toward Best Practice in Livestock Microbiota Research: A Comprehensive Comparison of Sample Storage and DNA Extraction Strategies

**DOI:** 10.3389/fmicb.2021.627539

**Published:** 2021-02-23

**Authors:** Gertrude Wegl, Nikolaus Grabner, Andreas Köstelbauer, Viviana Klose, Mahdi Ghanbari

**Affiliations:** BIOMIN Research Center, Tulln an der Donau, Austria

**Keywords:** livestock microbiota, next generation sequencing, DNA extraction, sample storage, 16S rRNA gene

## Abstract

Understanding the roles of microorganisms in the animal gastrointestinal microenvironment is highly important for the development of effective strategies to manage and manipulate these microbial communities. In order to guide future animal gut microbiota research projects and standardization efforts, we have conducted a systematic comparison of 10 currently used sample preservation and DNA extraction approaches for pig and chicken microbiota samples and quantified their effects on bacterial DNA yield, quality, integrity, and on the resulting sequence-based bacterial composition estimates. The results showed how key stages of conducting a microbiota study, including the sample storage and DNA extraction, can substantially affect DNA recovery from the microbial community, and therefore, biological interpretation in a matrix-dependent manner. Our results highlight the fact that the influence of storage and extraction methods on the resulting microbial community structure differed by sample type, even within the same species. As the effects of these technical steps are potentially large compared with the real biological variability to be explained, standardization is crucial for accelerating progress in the area of livestock microbiota research. This study provided a framework to assist future animal gut microbiota research projects and standardization efforts.

## Introduction

It is estimated that the global human population will reach 9 billion by 2050 (Silva, 2019). This constant growth of the human population is associated with a growing demand for food of both plant and animal origins. Meat production, especially in the pork and poultry sector, is therefore expected to increase from 338 million tons (2019) to 460 million tons by 2050, making a change in livestock production practices toward intensification and economic production necessary (Alexandratos and Bruinsma, 2012; Galketi et al., 2020). However, at the same time public demands for animal health and welfare are rising (Guarino et al., 2017). Meeting these challenges, the animal livestock sector, including veterinarians, nutritionists, feed manufacturers, breeders, and pharmaceutical companies, has a growing interest in animal microbiota research as the gastrointestinal microbiota modulates several important physiological functions such as digestion and absorption, energy metabolism, and immune system development, and helps in the prevention of pathogenic infections (Deusch et al., 2015; Marchesi et al., 2016; Zheng et al., 2020).

With the application of high-throughput next-generation sequencing (NGS) technologies, our understanding of the livestock microbiota, including the gut microbiota, has been greatly improved. NGS has enabled a relatively unbiased view of the overall composition and function of the animal gut microbiota, which is more accurate than traditional culture-based methods (Gupta et al., 2019; Zhou et al., 2019). While there are benefits of using NGS to assess the gut microbiota, experimental bias can be introduced during critical experimental steps (Choo et al., 2015; Panek et al., 2018). These key steps include selection of sample storage media, temperature and length of sample storage, the DNA extraction method, and region of the 16S rRNA gene amplified (in amplicon-based analyses), all of which may influence the results obtained (Rintala et al., 2017; Pollock et al., 2018; Zhou et al., 2019).

Information about the impact of overall experimental bias on the livestock gut microbiota is crucial for large-scale, time-series, and field microbiota analysis projects. Sample integrity, therefore, becomes an issue, as it is not logistically feasible for researchers to collect and process samples on the same day, and in many cases freezing at −80°C (the recommended storage temperature for microbiota material) is not possible. Instead, samples are usually taken at various time points and are collected and stored for future analysis. Consequently, a good method for sample storage, including both storage media and storage time, and a subsequent compatible DNA extraction protocol is essential for downstream NGS to accurately recover the gut microbiota (Zhou et al., 2019).

**FIGURE 1:**
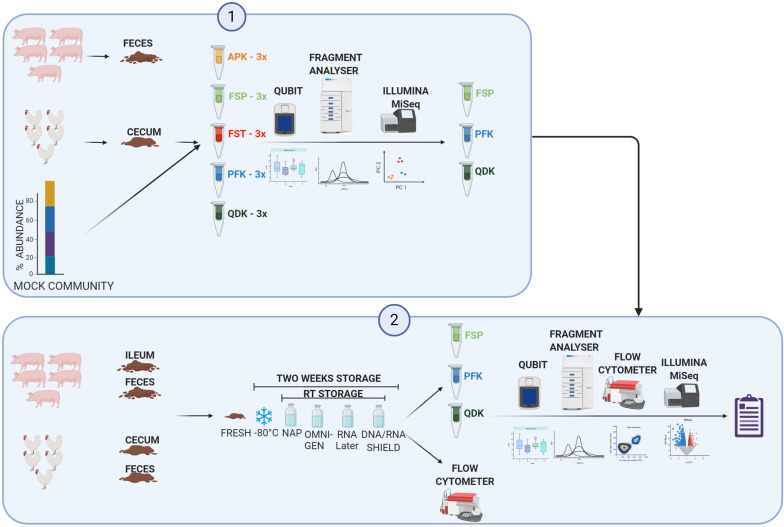
Diagram of experimental design. Phase 1: Gut digesta was collected from five healthy animals and pooled. Along with a standard mock community, pooled gut digesta samples were subjected to DNA extraction using five commonly used DNA extraction kits [the Allprep PowerFecal DNA/RNA Kit (APK), the QIAamp PowerFecal kit (PFK), the MP Biomedicals FastDNA Spin kit (FSP), the QIAamp Fast DNA Stool Mini kit (FST), and the Zymo Quick-DNA Fecal/Soil Microbe Kit (QDK)]. The extracted DNA was subjected to a quality check, integrity analysis, and Illumina sequencing of the 16S rRNA gene. Phase 2: Gut digesta were collected from five healthy animals, pooled, homogenized, and stored in any of the four storage media either at room temperature (RT) or at −80°C for 2 weeks. Samples were then subjected to DNA extraction via three DNA extraction kits, which were selected based on the results of Phase 1. Extracted DNA was subjected to a quality check, DNA integrity analysis, and Illumina sequencing of the 16S rRNA gene. The total number of bacteria cells in each sample were quantified using flow cytometry, and the data was used to calculate the quantitative microbiome profiling (QMP) of the samples (details are provided in the “Materials and Methods” section). Created with BioRender.com.

Herein, we compared the impact of storage media and DNA extraction methods on the recovery of gut microbiota from chickens and pigs (Figure 1). To ensure that the findings were applicable to the real world, samples from different gut sections were investigated including cecum digesta and colon digesta from chicken as well as ileum and feces from pigs. To the best of our knowledge, the present study is the first to systematically assess the effect of the current sample storage and DNA extraction approaches and their interactions on the chicken and pig gut microbiota. Importantly, the findings of this study are relevant for livestock microbiota analysis efforts because there is a paucity of data regarding the effects of the sample processing methodologies on the animal gut microbiota.

## Materials and Methods

### Sample Collection

Different sample types from pig and chicken were obtained from conventional slaughterhouses for each of the experimental phases. Subsequent to the euthanasia of the animals, the gastrointestinal tract was removed, stored at 4°C and immediately transported to the lab. For collection of intestinal content and feces (sampled from the distal part of the large intestine and herein after referred to as feces), samples from five animals were pooled and homogenized. Figure 1 shows an overview of the study design.

### Phase I–Evaluation of DNA Extraction Methodologies

Chicken cecum and pig feces were subjected to each of the defined extraction procedures in three replicates. To assess for bias in extraction, commercially available mock community controls were used. This mock community contains eight bacteria with the same abundance: *Staphylococcus aureus*, *Enterococcus faecalis*, *Listeria monocytogenes*, *Bacillus subtilis*, *Salmonella enterica*, *Lactobacillus fermentum*, *Escherichia coli*, and *Pseudomonas aeruginosa*, and two yeast species, with the same abundance: *Saccharomyces cerevisiae* and *Cryptococcus neoformans*. We used two types of these controls: (1) mock community microbes (ZymoBIOMICS Catalog #D6300), which used 75 μL aliquots as starting material for each of the DNA extraction kits; and (2) commercial mock community DNA (ZymoBIOMICS Catalog #D6305); this was used as the input DNA for library prep and subsequent sequencing run for this study. For each extraction method, we included a negative reagent-only control where an empty lysing tube was used to assess the contamination present in extraction reagents or because of the extraction protocol. The following DNA isolation procedures were examined, namely, the Allprep^®^ PowerFecal DNA/RNA Kit (QIAGEN, Germany), the FastDNA^TM^ Spin kit for Soil (MP Biomedicals^TM^, United States), the QIAamp^®^ Fast DNA Stool Mini kit (QIAGEN, Germany), the QIAamp^®^ PowerFecal^®^ Kit (QIAGEN, Germany), the Quick-DNA^TM^ Fecal/Soil Microbe Kit (Zymo Research, United States). For each sample type and each extraction kit, DNA extraction was performed in replicates from three separate aliquots. The initial starting material was 100 mg biomass for all extraction kits. If no mechanical lysis step (bead beating or vortexing) was included in the original protocol, bead beating was applied. For all extraction kits where bead beating was recommended, the Precellys was used at 5000 rpm for 20 s. Finally, DNA was eluted in 50 μL of Tris–HCl (pH 8) buffer.

#### Allprep PowerFecal DNA/RNA Kit (APK)

DNA isolation was performed according to the manufacturer’s instructions, with minor modifications. For microbial lysis, the microbial lysis tubes were vortexed at maximum speed for 5 min. Before the final washing steps, column digestion of RNA was performed as suggested in the protocol.

#### The FastDNA Spin Kit for Soil (FSP)

DNA isolation was performed according to the manufacturer’s instructions, with minor modifications. The Tris–HCl buffer was incubated at 55°C for 5 min before elution of the DNA.

#### QIAamp Fast DNA Stool Mini Kit (FST)

DNA isolation was performed according to the manufacturer’s instructions, with minor modifications. Because no mechanical lysis was included in the original protocol, bead beating was performed using the Precellys Glass kit 0.1 mm beads. During the additional heat lysis, the temperature was increased to 95°C.

#### QIAamp PowerFecal DNA Isolation Kit (PFK)

DNA isolation was performed according to the manufacturer’s instructions.

#### Quick-DNA Fecal/Soil Kit (QDK)

DNA isolation was performed according to the manufacturer’s instructions, with minor modifications. Since no clear details for mechanical lysis were provided, beat beating was performed.

### Phase II–Evaluation of Sample Storage Methodologies

Fresh biomass from chicken cecum and feces as well as pig ileum and feces were aliquoted for DNA extraction by FST, PFK, and QDK in three aliqots, under the following test conditions (Figure 1): freshly extracted (F), frozen at −80°C for 2 weeks (M), resuspended in a 10× volume of the stabilization reagents RNALater (Thermo Fisher Scientific, United States), DNA/RNA Shield (Zymo Research, United States), and a homemade nucleic acid preservation buffer (NAP) (Camacho-Sanchez et al., 2013), or use of the OMNIgene-GUT stabilization kit (OMR-200; DNA Genotek, Canada) and stored at ambient temperature for 2 weeks. In addition to DNA extraction negative reagent-only control, a blank sample containing only the storage buffer including RNALater^®^, DNA/RNA Shield, NAP, and OMNIgene-Gut was used to assess the contamination present in the buffers.

### DNA Quantitation and Quality Assessment

Subsequent to DNA isolation, DNA concentrations were measured using the Qubit^TM^ dsDNA BR assay kit on a Qubit^TM^ 2.0 fluorometer (Invitrogen^TM^, United States). DNA purity was determined by measuring ratios of absorbance at 260/280 and 260/230 using a spectrophotometer (NanoDrop^®^ ND-1000; Thermo Fisher Scientific, United States), while DNA shearing was evaluated using the High Sensitivity Large Fragment 50 kb Analysis kit on the Fragment Analyzer^TM^ (Agilent Technologies, United States).

### Microbial Community Structure Analysis by 16S rRNA Gene Amplicon Sequencing

DNA samples (including 25 negative controls) were sent for sequencing to Microsynth Austria GmbH for 16S rRNA gene amplicon sequencing using Nextera two-step PCR amplification using the primer set 341F_ill/802R_ill (V3–V4 region) (Klindworth et al., 2013) and equimolar pooling for library preparation. The obtained amplicon libraries were sequenced on the Illumina MiSeq system using the paired end protocol [300 bp, paired-end read (PE), V3 chemistry] according to the manufacturer’s instructions.

### Flow Cytometry Analysis

Aliquots of chicken and pig samples were stored as described above for phase II. Fresh samples were used immediately for cell count determinations, and the remaining samples were stored in the DNA stabilizing reagent or at −80°C for 2 weeks until analysis. For cell counting, 100 mg aliquots were suspended in 10 ml of physiological saline by stirring, and then centrifuged at 400 × *g* for 5 min. After centrifugation the supernatant (which was considered to be a 1:100 dilution) was diluted further in a 1:10 dilution series to a range that allowed measurement (as determined in pre-experiments). Next, 1 mL of the microbial cell suspension was stained with 1 μL SYBR^®^ Green I (1:100 dilution in dimethyl sulfoxide; shaded 10 min incubation at 37°C; 10,000 concentrate; Thermo Fisher Scientific, United States) and immediately measured on a BD Accuri C6 flow cytometer (BD Biosciences, United States). Fluorescence events were monitored using FL1 533/30 nm and FL3 > 670 nm optical detectors.

### Sequence Data Processing and Statistical Analysis

Sequenced libraries were demultiplexed in MiSeq^TM^ Reporter v2.6, and FASTQ files were processed using USEARCH v.10.0.240 (Edgar, 2013). FASTQ sequences were stitched and filtered to the approximate size of the V3–V4 region of the bacterial 16S rRNA gene. USEARCH was used to trim primer regions and remove chimeric and low-quality sequences. Sequences were filtered with the maximum expected errors per sequence ≤0.5. These cutoffs followed default or more stringent parameters as outlined in the USEARCH Guide^1^. Filtered reads were subsequently denoised, pre-clustered by size and then clustered into operational taxonomic units (OTUs) (99% similarity) using the UNOISE algorithm (Edgar, 2017). A 0.002% minimum relative abundance filter was applied to the OTU table, deleting extremely low-abundance counts that have a higher probability of being spurious. All of the sequenced negative control samples resulted in fewer than 50 reads per samples, and therefore, excluded from downstream analysis (see Supplementary Data File 1 which contains detailed information on the samples including library size). The R-packages phyloseq (v. 1.30.0) (McMurdie and Holmes, 2013), vegan (Oksanen et al., 2016) and DESeq2 (Love et al., 2014) were used for microbiota data handling and calculating alpha (observed richness and Shannon index) and beta diversity as well as differential abundance analyses. Where applicable, features with a false discovery rate (FDR) of less than 10% were considered significant.

For quantitative microbiome profiling (QMP), the matrix was built as described by Vandeputte et al. (2017). In brief, samples were downsized to an even sampling depth, defined as the ratio between sampling size (16S rRNA gene copy number-corrected sequencing depth) and microbial load (the average total cell count per gram of digesta material; Supplementary Data File 1). 16S rRNA gene copy numbers were retrieved from the rRNA operon copy number database, rrnDB (Klappenbach et al., 2001). The copy number-corrected sequencing depth of each sample was rarefied to the level necessary to equate the minimum observed sampling depth in the cohort. Rarefied bacterial abundances were converted into cell numbers per gram. The R code used to compute QMPs can be found at https://github.com/raeslab/QMP/.

For analysis of DNA yield, quality, and alpha diversity in the first and second phase, a non-parametric Wilcoxon test was used to compare the effects of the isolation kits as well as the storage approaches, stratified by sample type. Additionally, for the second phase, a linear regression was performed where the predictors were storage type, extraction method, and an interaction term between storage type and extraction method to determine if the impact of extraction method on the final outcomes varied by storage type, followed by a pairwise analysis.

## Results

### DNA Extraction Outcome Is Kit and Matrix Dependent

**FIGURE 2:**
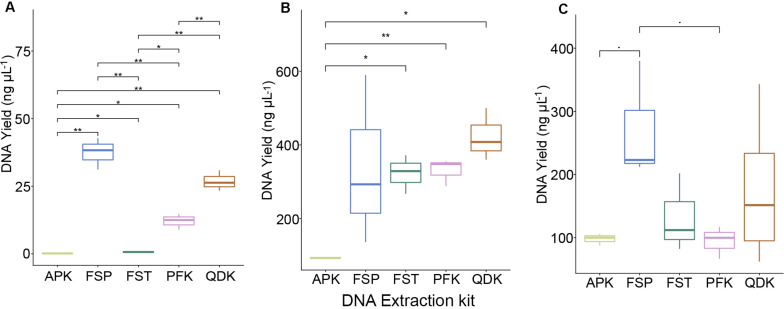
DNA yield of DNA extractions from whole cells and intestinal material. DNA was extracted from a mock community and livestock samples using five commercial DNA extraction kits. Boxplots show the DNA yield (ng μL^−1^) after extracting the pure mock community **(A)**, pooled samples from chicken cecum **(B)**, and pig feces **(C)**. Values represent averages from triplicate DNA extractions. Significant differences were tested with the Kruskal–Wallis test, which were FDR corrected, with *P < 0.05, **P < 0.01, and ^-^*P* < 0.09.

Depending on the DNA extraction kit applied, DNA concentrations varied greatly across all sample types (Figure 2). On average, the MP Biomedicals FastDNA^TM^ Spin kit (FSP) and Zymo Quick-DNA^TM^ Fecal/Soil Microbe Kit (QDK) resulted in the highest DNA yields (mock community >25 ng μL^–1^, chicken cecum >340 ng μL^–1^, pig feces >175 ng μL^–1^), although significant differences in the remaining DNA extraction kits were mainly observed when DNA was extracted from the whole cell mock community (Figure 2A). The QIAamp^®^ Fast DNA Stool Mini kit (FST) (except in the mock community extractions) and the QIAamp^®^ PowerFecal^®^ kit (PFK) were mid-performers among our five tested kits, with alternating ranking between them depending on the sample type; in mock community extractions, FST yielded low DNA quantities (Figure 2A). The Allprep^®^ PowerFecal DNA/RNA Kit (APK) always yielded low concentrations of DNA (mock community average <1 ng μL^–1^, livestock sample average <100 ng μL^–1^). Of DNA purity (260/280 ratio), most of the samples were within the expected range, and only samples with very low DNA yields, such as those extracted by APK, did not meet the quality criteria (260/280 value within 1.8–2.0; see Supplementary Data File 1).

**FIGURE 3:**
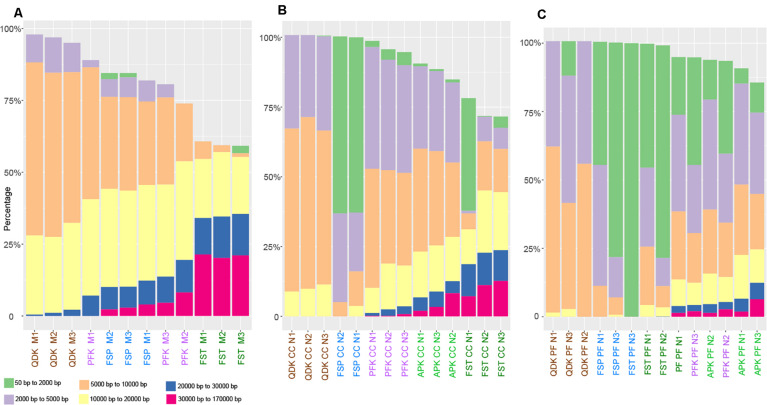
Fragmentation profiles of DNA extracted by different isolation kits. Mock community DNA **(A)**, as well as DNA extracted from chicken cecum **(B)** and pig feces **(C)** was subjected to analysis on an Agilent Fragment Analyzer System. The different user defined size classes are plotted as stacked bars representing % of total DNA. Samples with DNA concentrations below 0.4 ng μL^–1^ were excluded from the analysis.

As an additional measure of DNA quality, DNA integrity was determined on a Fragment Analyzer System (Agilent, Santa Clara, CA, United States), resulting in a length profile of the extracted DNA fragments for each sample. There was considerable variation in the fragmentation of the obtained DNA depending on the DNA extraction kit used; however, the sample type had a major influence (Figure 3). Within the livestock samples (chicken cecum and pig feces), especially in the pig-derived fecal matrix, a high proportion of small-sized DNA fragments (<5,000 bp) was obtained. In the mock community and chicken cecum, the FST was able to extract high quality DNA, for which more than 30% of the total DNA extracted with the FST consisted of fragments larger than 10,000 bp; however, this same kit produced heavily sheared DNA in pig feces extractions. Aside from the FST, the highest proportions of high molecular weight (HMW) DNA (>10,000 bp) were obtained using the APK (no data for mock community) and PFK, and the majority of the fragments from these two kits were between 2,000 and 20,000 bp. Using the QDK and FSP, the majority of the DNA fragments were below 10,000 bp (Figure 3).

### DNA Extraction Strategies Failed to Recapitulate the Exact Composition of the Mock Community

For the phase 1 of study a total of 1,807,438 high quality 16S rRNA sequence reads (90% of the raw reads) with the average of 35,439 (±15,517 SD) PE reads per sample (*n* = 51) were used as inputs for downstream analysis (see Supplementary Data File 1 which contains detailed information on the samples including library size).

**FIGURE 4:**
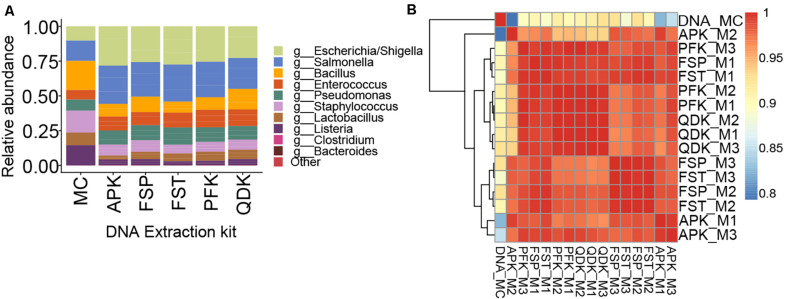
Sequencing results for extractions of a whole cell mock community by five different methods. To control for biases during sequencing, a mock DNA standard was also included in sequencing and analysis of mock community (whole cell) extractions. Within the relative abundance analysis **(A)**, the top 10 genera are shown for comparison. Species that were not present in more than 10% of the samples were removed. Heatmap showing the Spearman correlation coefficient of pairwise comparison between the samples **(B)**. The color of the squares indicates the level of correlation. Red squares indicate large correlations; blue squares indicate small correlations. Triplicate extractions with each technique were performed; sample names indicate the extraction technique followed by M1, M2, or M3, indicating each of the triplicate mock community extractions. DNA_MC indicates the mock community DNA standard.

By incorporating a standard microbial community into the experiment, we aimed to gain better insight into the potential biases introduced by the extraction method on microbial community composition (i.e., membership) and structure. The relative abundances of 16S rRNA gene sequences for various microbial taxa resulting from NGS of DNA extracted from a mock microbial community using five different commercially available extraction kits are shown in Figure 4. As can be seen, none of the applied extraction kits reproduced an exact profile of the mock community DNA (Figure 4A). In general, smaller proportions of the Gram-positive genera *Listeria*, *Lactobacillus*, and *Staphylococcus* were obtained, although there was only a minor effect on *Enterococcus*, whereas the proportion of the Gram-negative genera *Escherichia* and *Salmonella* was over-represented in the samples extracted using the tested extraction kits. In our evaluation of the mock microbial community results for extracted DNA from each extraction method, we found that the mock community resulting from DNA extracted using the PFK and QDK had the highest Spearman’s rank correlation coefficients (ρ > 0.90) to the composition of the mock community DNA standard and the greatest consistency among replicates, suggesting that the PFK and QDK most closely approach the approximated composition of the mock community (Figure 4B). Given that we had only one replicate for the mock DNA standard (Figure 4B), it was not possible to perform statistical analyses on the effect of the extraction method on differential abundance in the mock communities.

### DNA Extraction Method Deeply Influences the Pig and Chicken Gut Microbiota Profile

**FIGURE 5:**
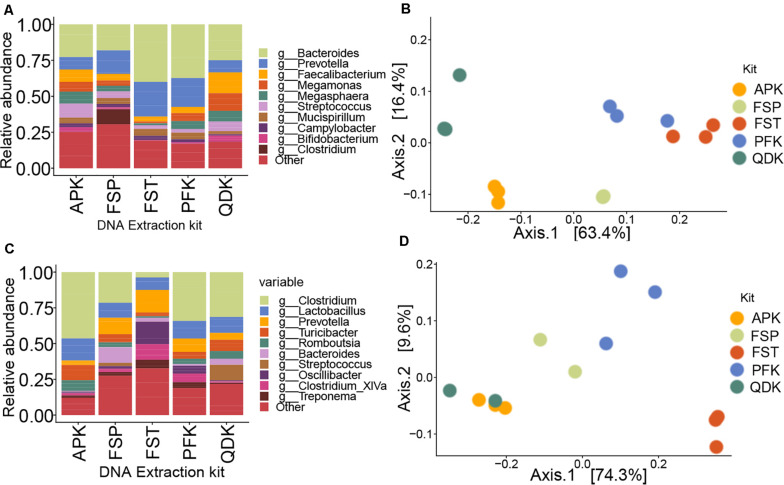
Performance comparisons among DNA isolation kits in 16S rRNA gene sequencing of livestock samples. Within the relative abundance analysis, the top 10 genera are shown for comparison of pooled samples from chicken cecum **(A)** and pig feces **(C)**. Species that were not present in more than 10% of the samples were removed. Principal coordinate analysis (PCoA) plot based on Bray–Curtis dissimilarity was used to examine the dissimilarities between microbial communities in the chicken- **(B)** and pig- **(D)** derived samples.

The relative abundances of the top detected genera in each sample, grouped by their extraction method, are displayed in Figure 5. Chicken cecum was characterized by a high proportion of Gram-positive bacteria when extracted with the APK, FSP, or QDK kits, whereas the FST and PFK revealed a high abundance of the Gram-negative genera *Bacteroidetes* and *Prevotella* (Figure 5A). Only the FSP kit was able to extract a major proportion of *Clostridium* DNA. Pig feces were generally dominated by the phylum Firmicutes (Figure 5C); however, differences in the relative abundances of genera within this phylum were observed among extracts from the different kits. Comparing the microbial profiles resulting from extracts of the different kits, the FST kit revealed a remarkably high proportion of the genera *Oscilibacter* and *Clostridium XIVa*, while the relative abundance of the genus *Clostridium* was reduced.

The impact of the extraction method on the detected microbial community structure is shown in Figures 5B,D, which depicts a Principal Coordinates Analysis (PCoA) based on Bray–Curtis dissimilarity. It is important to point out that, because we analyzed livestock samples, the true diversity, composition, and structure of the sampled communities are unknown. Therefore, we cannot confidently determine which kit most reliably reproduced the original microbial profile. In fact, our aim here was to determine how the general microbial profiles were similar or different among the different extraction kits. If two or three different strategies resulted in similar profiles, we assumed that those methods provided a closer representation of the actual microbial community than methods displaying different clustering. Multivariate statistical analysis performed using a permutational analysis of variance (PERMANOVA) showed that extraction method accounted for 50% of the variability (*P* = 0.005) in microbial community composition in chicken cecum samples and 45% of the variability (*P* = 0.05) in pig feces samples. For chicken cecum microbial profiles, profiles produced from PFK and FST extracts clustered closely together, but microbial profiles from APK, FSP, and QDK extracts formed distinct clusters that were widely separated from each other along the PCoA axes (Figure 5B). For pig feces, however, the profiles from FST extracts showed low similarity to those of the other extraction kits (Figure 5D). Alpha diversity analysis on the livestock samples based on the observed OTUs and Shannon index showed no significant effects of the extraction methods, except for pig feces samples where FST extraction resulted in significantly higher Shannon index values (FST: 6.27 ± 0.09, PFK: 5.86 ± 0.19, FSP: 5.50 ± 0.14, AP: 4.66 ± 0.16, QDK: 4.45 ± 0.43). As expected, pig feces samples showed a higher alpha diversity compared to the chicken cecum samples. In general, samples extracted using the FSP kit showed the highest observed index (pig feces: 2006 ± 517, chicken cecum: 1548 ± 139), while those extracted by QDK showed the lowest (pig feces: 1597 ± 311, chicken cecum: 1307 ± 367), almost regardless of the sample type. The observed index for the rest of the extraction kits were as follow: APK (pig feces: 1851 ± 179, chicken cecum: 1570 ± 15), FST (pig feces: 2108 ± 91, chicken cecum: 1451 ± 84), PFK (pig feces: 1942 ± 280, chicken cecum: 1484 ± 258).

### DNA Extraction, Sample Storage, and Their Interaction Affect the Recovery and Integrity of DNA

In a secondary analysis examining the impact of sample storage on DNA yield, we focused on three DNA extraction kits, FST, PFK, and QDK, which were selected based on their DNA yield, purity, and integrity results in Phase 1, as well as the extent to which these kits were able to reliably reproduce mock community profiles (Figure 1).

**FIGURE 6:**
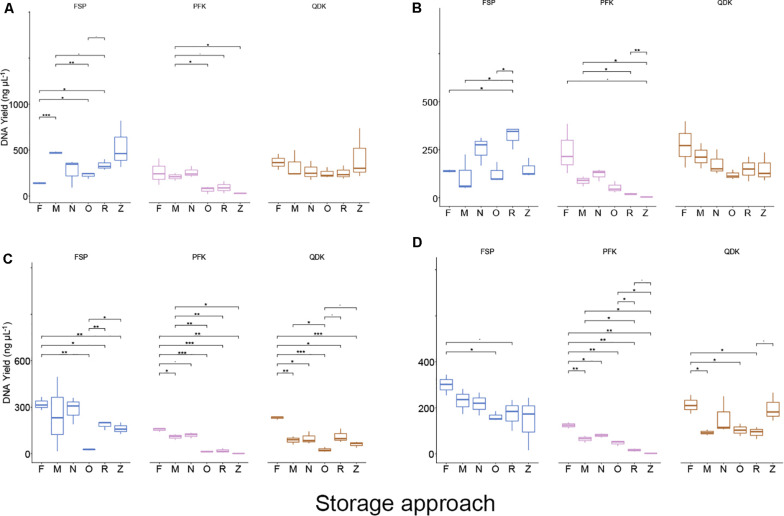
DNA yields from livestock intestinal and fecal samples using different storage conditions and isolation kits. Boxplots showing the DNA yield (ng μL^−1^) after extracting the livestock bacterial community from chicken cecum **(A)**, chicken feces **(B)**, pig ileum **(C)**, and pig feces **(D)**. Values represent averages from triplicate DNA extractions. Kruskal–Wallis test FDR corrected, *P < 0.05, **P < 0.01, ***P < 0.001, and ^-^*P* < 0.09, respectively.

Using linear regression, to assess the impact of extraction method, storage, and their interaction on DNA concentration in each sample matrix, both sample storage and DNA extraction methods were predictors of DNA yield (*P* < 0.001, with the exception of chicken cecum samples for which *P*_*storage effect*_ = 0.07). Analyses adjusted for the storage media effect demonstrated that for the chicken (cecum and feces) samples, the FSP and QDK kits produced significantly (*P* ≤ 0.001) higher DNA yields, whereas the PFK resulted in rather low DNA yields from chicken (cecum and feces) and pig samples (ileum and feces) (Figure 6).

With regard to storage, a mild effect of storage strategies on the DNA yield was observed in chicken cecum samples, where storage at −80°C, Zymo DNA/RNA Shield, and NAP buffer improved DNA yield relative to the freshly extracted samples (Figure 6). For the chicken feces samples, storing the samples in OMNIgene-GUT buffer, ZYMO DNA/RNA Shield, or at −80°C, resulted in significantly lower DNA yield compared to yields from either freshly extracted samples (O:P = 0.003, Z:P = 0.004, M:P = 0.04) or samples stored in NAP buffer (*P* < 0.05). Freshly extracted pig ileum and pig feces samples showed significantly higher DNA yields (*P* ≤ 0.001) compared to other storage strategies (Figure 6). Pig ileum and feces samples stored in NAP buffer or those stored at −80°C for 2 weeks showed significantly (*P* < 0.05) better performance in DNA yield than the other tested sample storage methods, yet these yields were still significantly lower than those of freshly extracted samples (Figure 6).

According to the findings of our linear regression analysis, the interaction term between the extraction method and storage approaches was statistically significant for the chicken cecum and feces samples (*P* = 0.001 and *P* = 0.009, respectively), indicating that there was no single extraction method that had the highest DNA yield for all sample types stored differently (Figure 6). For the pig-derived samples, however, the interaction term was not statistically significant (*P* = 0.1), suggesting that one DNA extraction methodology (FSP) resulted in a higher yield of DNA, regardless of the storage type. Concerning DNA quality (260/280 ratio), most of the samples were within the expected range, with some exceptions, specifically chicken fecal samples that were freshly extracted with the FSP kits and PFK extracts of chicken feces, pig ileum, and pig feces stored in either RNALater or DNA/RNA Shield (Supplementary Data File 1).

**FIGURE 7:**
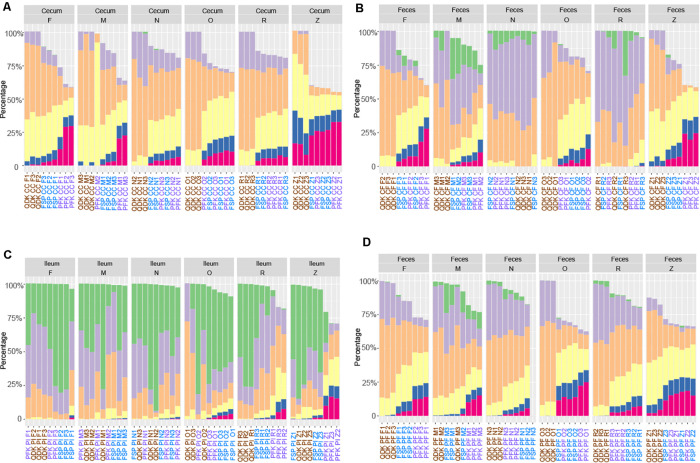
Effect of sample storage on DNA fragmentation profiles using different isolation kits. Chicken cecum **(A)** and feces **(B)** DNA, as well as DNA extracted from pig ileum **(C)** and feces **(D)** was subjected to analysis via the Fragment Analyzer. The different user-defined size classes are plotted as stacked bars representing % of total DNA. Samples with a DNA concentration below 0.4 ng μL^–1^ were excluded from the analysis.

Storage strategy had a strong effect on the integrity of the extracted DNA from the chicken and pig microbiota samples (*P* < 0.001), with the exception of pig feces, for which the extraction kit showed a more significant effect (*P* < 0.01). Linear regression analysis showed that regardless of the matrix, while adjusting for DNA extraction kit, samples stored at DNA/RNA Shield showed a significantly higher percentage of HMW, (i.e., >30 kb) DNA fragments in comparison to fresh samples (chicken cecum: *P* < 0.001, chicken feces *P* = 0.4, pig ileum: *P* < 0.001, pig feces: *P* < 0.005) as well as other storage approaches (*P* < 0.01, Figure 7). For the pig feces samples stored in OMNIgene-GUT buffer, a significantly higher abundance of HMW DNA was observed in comparison to fresh samples (*P* = 0.003) as well as those stored in other media (*P* < 0.001, Figure 7). In line with observations from the first phase, regardless of the matrix, the PFK extraction kit provided a significantly higher percentage of HMW DNA (*P* ≤ 0.01), followed by FSP extraction kit (*P* < 0.01), for the chicken cecum and pig feces samples.

### Storage Media but Not Extraction Method Has the Biggest Impact on the Pig and Chicken Gut Microbial Profile

Illumina sequencing of the samples analyzed in phase 2 of the current study (*n* = 245) produced a total of 20,515,029 PE reads, with an average of 83,734 sequences per sample (±55,338 SD), post-quality filtering (Supplementary Data File 1).

**FIGURE 8:**
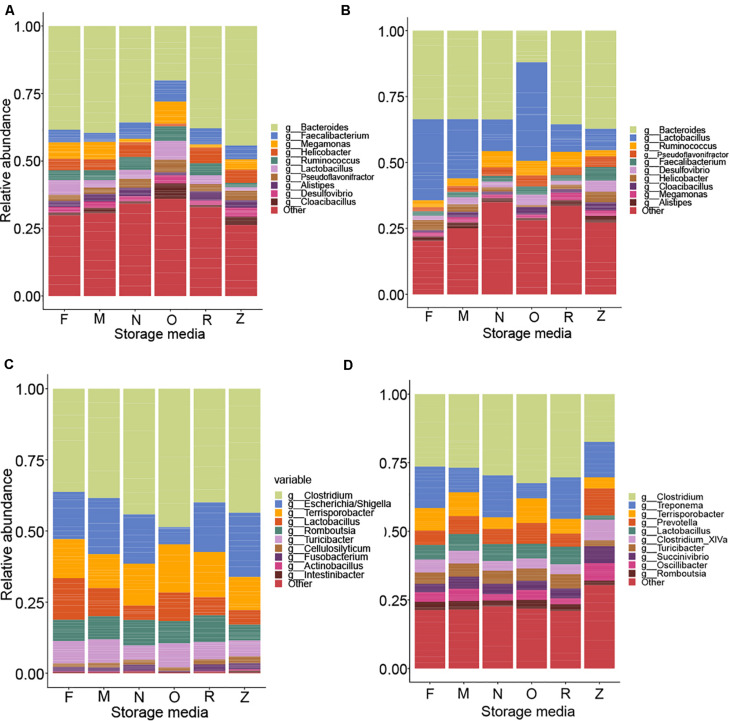
Comparisons of different storage media on 16S rRNA gene sequencing of livestock samples. Within the relative abundance analysis, the top 10 genera are shown for comparison for pooled samples collected from the chicken cecum **(A)**, chicken feces **(B)**, pig ileum **(C)**, and pig feces **(D)**.

**FIGURE 9:**
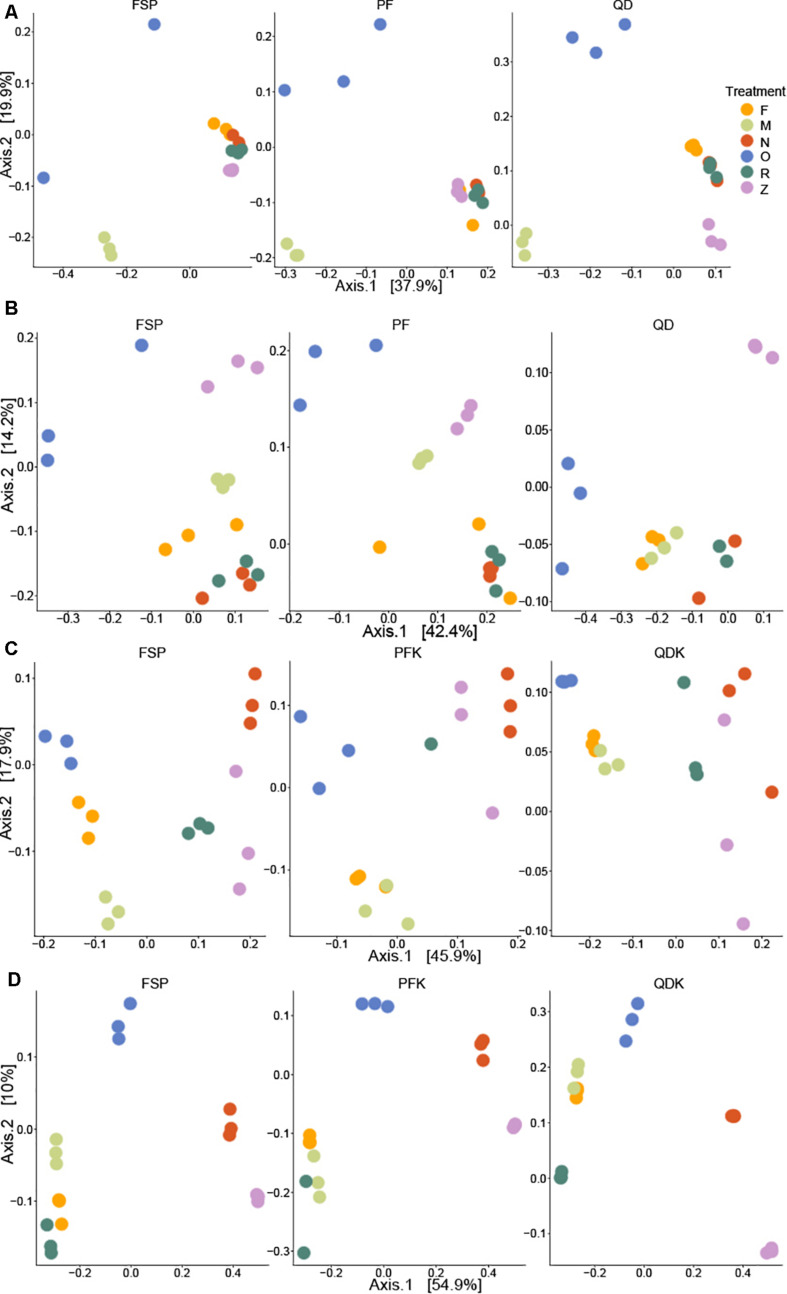
Principal Coordinates Analysis (PCoA) of differences in beta diversity among samples. Chicken cecum **(A)**, chicken feces **(B)**, pig ileum **(C)**, and pig feces **(D)** samples. Bray–Curtis dissimilarity was used to calculate beta diversity. Each dot represents one sample and each color represents the way the sampled was stored prior to extraction by each of the three DNA extraction kits. F: freshly extracted. M: frozen at −80°C, N: NAP buffer, O: OMNIgene-GUT buffer, R: RNALater, Z: ZYMO DNA/RNA Shield. Samples with more similar microbial community profiles cluster together, whereas those with more dissimilar microbial profiles are a further distance apart.

The relative abundances of the top genera of the bacteria found in each sample are depicted in Figure 8. Given that storage strategies had a profound effect on the integrity of DNA, we assumed that this impact could be due to the lysis of the microbial cells during storage in the storage buffers. Indeed, by using flow cytometry, we observed up to a five-fold variation in cell counts among samples stored in different buffers, samples stored frozen, and fresh samples (Supplementary Data File 1). To account for this variation in the effect of storage approaches on the chicken and pig gut microbiota, a QMP technique was used by combining amplicon sequencing and flow cytometry data (Vandeputte et al., 2017). In brief, the QMP technique adjusts read matrices for differences in sampling depth (that is, it standardizes them to equal amounts of sequencing data per cell in a gram of the original samples) and then extrapolates the resulting rarefied taxon abundances to a sample’s total microbial load. By doing so, QMP reduces both false-positive and false-negative rates in downstream analyses, lowering the risk of erroneous interpretation of microbiota associations (Vandeputte et al., 2017). The impacts of the extraction and storage methods on the detected microbial community structure are shown in Figure 9, which depicts a PCoA based on Bray–Curtis dissimilarity.

Permutational analysis of variance based on Bray–Curtis dissimilarity was performed for statistical testing of the effects of sample storage, DNA extraction, and their interaction on the observed microbial community structure. With the exception of the pig feces samples, where the extraction kit accounted for the majority of the variability in the microbial community structure, storage approaches were the major driver of microbial community structure in chicken and pig samples (*R*^2^ storage chicken cecum = 0.50, *R*^2^ storage chicken feces = 0.54, *R*^2^ storage pig ileum = 0.37, and *R*^2^ storage pig feces = 0.33).

**FIGURE 10:**
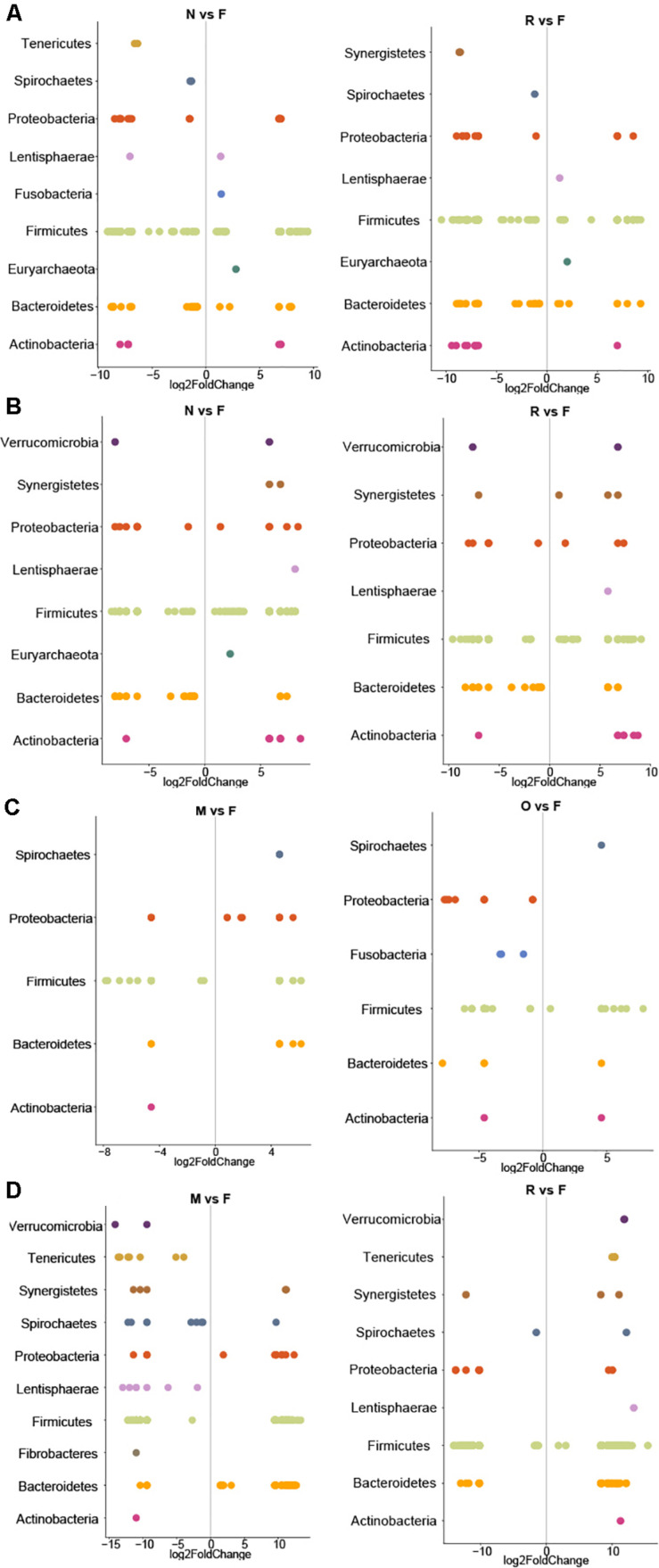
Impact of extraction method on differential abundances of microbiota. Significant (*q* < 0.05) log-fold changes in the abundances of microbial phyla in samples stored at −80°C or in different storage buffers for chicken cecum **(A)**, chicken feces **(B)**, pig ileum **(C)**, and pig feces samples **(D)**. Positive log-fold change indicates an increase in abundance, while negative log-fold change indicates a reduction in abundance over time compared to the freshly extracted samples group in chicken cecum, chicken feces, pig ileum, and pig feces samples. F: Freshly extracted, M: frozen at −80°C, N: NAP buffer, O: OMNIgene-GUT buffer, R: RNALater, Z: ZYMO DNA/RNA Shield. Only the results for the storage condition that resulted in lowest number of differentially abundant taxa are shown for each sample type. The complete figure showing the results for all storage media and the full list of differentially abundant taxa with taxonomic resolution to the genus and species level can be found in Supplementary Data File 1 and Supplementary Figure 1.

**FIGURE 11:**
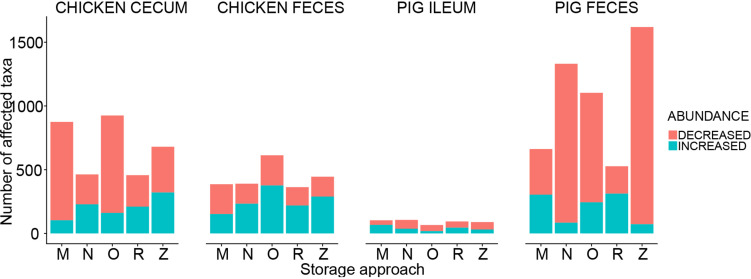
Impact of storage approaches on differential abundances of microbiota. The bar plot shows the number of affected taxa (either increased or decreased) based on differential abundance analysis in chicken cecum, chicken feces, pig ileum, and pig feces samples. M: frozen at −80°C, N: NAP buffer, O: OMNIgene-GUT buffer, R: RNALater, Z: ZYMO DNA/RNA Shield.

Adjusted for the effect of DNA extraction, the effect of storage methods on the differential abundances of microbial taxa among all samples is depicted in Figures 10, 11 (see Supplementary Data File 1 and Supplementary Figure 1 for further details). Microbial species with an FDR < 5% in the linear models were considered significant. In chicken cecum and feces, most of the differentially abundant OTUs belonged to the phylum Firmicutes, followed by Bacteroidetes and Proteobacteria (Figure 10). In line with the PCoA plot (Figure 9), the microbiota of the chicken cecum and feces samples stored in NAP buffer and RNALater showed the lowest number of differentially abundant taxa compared to the freshly extracted samples, resembling a more similar microbial profile (Figure 11). Storing the samples at −80°C or in OMNIgene-GUT buffer for 2 weeks caused an almost exclusive reduction in the abundances of Firmicutes, Bacteroidetes, Proteobacteria, and Tenericutes in the stored chicken cecum compared to freshly extracted samples (Figure 10); consequently, a more dissimilar microbial community profile to that of the freshly extracted samples was observed (Figure 9). In chicken fecal samples stored in OMNIgene-GUT buffer and DNA/RNA Shield, significant enrichment of Proteobacteria and Synergistetes was observed (Figure 10). The impact of storing the samples at −80°C or in OMNIgene-GUT buffer is obviously matrix dependent, since pig ileum samples stored frozen or in OMNIgene-GUT buffer showed the most similar microbial community structure to that of freshly extracted samples compared to those of the other storage approaches, almost regardless of the DNA extraction kit (Figures 8, 9).

Regardless of the DNA extraction kit used, PCoA analysis revealed a distinct clustering of the pig feces samples stored in DNA/RNA Shield or NAP buffer, suggesting a systematic difference between the microbial profiles of samples stored in these two preservative buffers and those of freshly extracted samples (Figure 9). Differential abundance analysis revealed that storing pig feces samples in either DNA/RNA Shield or NAP buffer resulted in the highest number of differentially abundant affected taxa and exclusive reduction in Bacteroidetes, Proteobacteria, Spirochaetes, Synergistetes, Lentisphaerae, Euryarchaeota, and other phyla, compared to freshly extracted samples (Figures 10, 11).

## Discussion

As the livestock microbiota field tends toward ever-larger data sets, increased standardization of techniques and dissemination of methods with low noise and bias will greatly increase the ability to deliver on the promise of translatability from small-scale studies to the field or natural environment. In order to guide future animal gut microbiota research projects and standardization efforts, a systematic comparison of 10 currently used preservation and DNA extraction approaches for pig and chicken-derived samples was performed. According to the findings, the sample storage approach significantly influenced the overall diversity and composition of the pig and chicken microbiota samples to a greater degree than the extraction method. Our results highlight the fact that the influence of sample storage and DNA extraction methods on the resulting microbial community structure differed by sample type, even within the same species, emphasizing the importance of using a consistent upstream sample processing protocol for all sample types in a single study.

Our findings extend the prior efforts in the field of livestock microbiota research in several ways. First, in order to ensure that the findings were applicable to real-world livestock microbiota research, regarding the effect of storage media and DNA extraction on the recovery of DNA and microbial community profiles, four widely used gut section/matrices from chicken and pig were selected. While we show that storage media and DNA extraction affect DNA yield and community composition in samples, the chicken cecum and feces samples were affected in the same way based on the analyzed parameters, indicating a highly similar microbial community profile (Stanley et al., 2015; Yan et al., 2019). On the other hand, pig ileum and pig feces samples showed a more heterogeneous response to the effect of the storage and DNA extraction methods, which highlights the profound difference in the community structure of the ileum and colon in pigs, as shown elsewhere (Crespo-Piazuelo et al., 2018). Second, one of our criteria used to evaluate each method relied on the measurement of DNA integrity/fragmentation using a fragment analyzer. In fact, the size of DNA fragments is a key parameter for NGS technologies as they rely on high-quality DNA that is suitable for library preparation followed by sequencing. This is even becoming more important given the recent advances in using third-generation sequencing technologies (e.g., MinION, PacBio) for microbiota studies that are strongly dependent on the size of DNA in the extracted samples. In line with some previous studies, we show, for example, that while the effect of storage and extraction methods differs by sample type on DNA integrity, the size of this modification was more pronounced due to the storage approach rather than extraction methodologies (Choo et al., 2015; Haj-Ahmad et al., 2015; Hill et al., 2016; Kia et al., 2016; Song et al., 2016; Greathouse et al., 2019; Zhou et al., 2019; Zymo Research, 2021). Finally, by implementing a novel approach, i.e., QMP, which integrates microbial cell counting into a sequencing workflow, we took into the account large differences in microbial loads among samples, resulting in lowering the risk of erroneous interpretation of microbiota associations.

Focusing on the first phase of the experiments, our results are generally consistent with aspects of prior literature focused on DNA extraction methods. Irrespective of the sample type, DNA isolation by the FSP and QDK resulted in the highest DNA yields. Similar observations/trends have been reported in other studies (Lu et al., 2015; Burbach et al., 2016; Ducarmon et al., 2020), although average concentrations might differ from published values due to differences in the experimental setup. Next to the bead-beating parameters (e.g., duration and speed), differences in bead types, buffer composition, and incubation temperature are known to affect the resulting DNA concentrations (Desneux and Pourcher, 2014; Ferrand et al., 2014; Knudsen et al., 2016) and might explain the increased lysis capacity of these kits. The APK kit revealed low DNA concentrations for both the mock community and livestock samples that would fail to meet the technical requirements for 16S rRNA gene sequencing or shotgun metagenomic sequencing on either NGS or third-generation platforms. This kit differed from the other kits evaluated in this study, as it was designed for the co-extraction of microbial DNA and RNA from fecal material, allowing the analysis of microbiota structure-function relationships without the need for additional extraction activities. Although this kit has been used in DNA-based NGS studies before (Cao et al., 2019; Haworth et al., 2019), no additional information from other comparative studies is available. Therefore, it can only be speculated that APK lysis conditions are mild compared to those of other kits, suitable for extraction of DNA and intact RNA, but not able to compete with the efficiency of extraction protocols focusing on DNA only, as stated elsewhere (Cuív et al., 2011). With regard to integrity, high DNA yields, as obtained by the FSP and QDK kit, were associated with an increased abundance of short DNA fragments. Besides the type and speed of the mechanical lysis procedure (vortexing or bead beating), there are also other factors such as type of beads, buffer composition, and incubation temperature, which might affect differences in DNA integrity (Videnska et al., 2019). Increasing the DNA yield is a key feature in selecting an extraction protocol; however, at the same time, fragmentation of DNA should not be ignored. Although the presence of HMW DNA is of major importance for third-generation sequencing technologies, protocols that consistently recover low-yield or highly fragmented DNA are likely to skew measured community composition (Costea et al., 2017; Lim et al., 2018). In fact, this could be the possible explanation for why none of the DNA extraction methods used in the first phase was able to precisely recap the known composition of the mock community (Figure 4). A general underestimation of Gram-positive bacterial abundance in the mock community was observed, which is most likely due to incomplete cell wall lysis of Gram-positive bacteria (Costea et al., 2017). Although these kits might be inefficient in extracting Gram-positive bacteria, however, one cannot conclude that the DNA from a particular bacterial family will be extracted preferentially using one specific DNA isolation method. Differences in cellular properties within members of the same genus or family can affect cell lysis (Sergeant et al., 2012; Burbach et al., 2016). Thus, it is not surprising that the microbial community structures of the livestock samples extracted by different kits varied, showing higher similarity of certain kits in one animal species, but a different clustering for another species (Figure 5). In fact, the chemical and physical composition of the sample matrix, such as the water content or the presence of certain compounds, such as humic acids and polysaccharides, can influence DNA isolation and downstream procedures (Rådström et al., 2004; Knudsen et al., 2016; Vandeputte et al., 2016).

Since immediate processing of samples is not possible under field conditions, rapid freezing at −80°C has become the gold standard for sample storage to stop the growth of residing bacteria or potential contaminants and to conserve microbial baseline abundances (Vandeputte et al., 2017). Nevertheless, freezing still does not guarantee full flexibility during sampling and transport, and it is often not logistically feasible. Freezing and thawing of bacterial cells might help in opening the cell envelope, making DNA extraction more efficient, yet it can compromise the integrity of the DNA (Chung et al., 2017). Thus, for the second phase of the experiments, we further investigated the effect of commonly used storage approaches on the DNA quality and the shift in the chicken and pig digesta microbial communities. In line with the previous literature, a general reduction in the DNA yield and integrity due to freezing was observed for the majority of the samples, although differences between frozen and freshly extracted samples were quite small compared to the effect of other storage approaches (Bahl et al., 2012; Wesolowska-Andersen et al., 2014; Metzler-Zebeli et al., 2016; Horng et al., 2018). This can explain the higher similarity of the microbial profile of the frozen samples compared to that of fresh samples.

An obvious matrix-dependent as well as interaction effect of the storage and DNA extraction methodologies was suggested by our findings. Constant low DNA yields obtained from samples stored in RNALater and DNA/RNA Shield and extracted by the PFK kit hint of a possible chemical incompatibility of the buffer and kit components. Since this reagent was also used to stabilize the commercially available whole-cell mock community, it also might explain the poor performance of kits in phase 1 of this study. This highlights the importance of checking the compatibility of the sample preservation buffer and extraction kit before starting a microbiota study (Hallmaier-Wacker et al., 2018; Chen et al., 2019).

For chicken samples, profiles obtained from samples stored in OMNIgene-GUT, a commercially available feces collection/stabilization system, diverged most from those of freshly extracted samples. Compared to profiles for the remaining storage conditions, microbial profiles of chicken microbiota samples stored in OMNIgene-GUT revealed almost twice the number of OTUs and showed significantly different abundances of microbial taxa, which was also reflected in the obtained alpha-diversity measures. Interestingly, several studies have validated the performance of OMNIgene-GUT in preserving the microbial composition in human fecal samples (Chen et al., 2019; Ilett et al., 2019; Lim et al., 2020) and this system revealed good to medium performance in stabilizing the pig microbiota in our study. As this reagent was initially developed for human intestinal microbiota (Doukhanine et al., 2016), it might therefore be more suitable for the mammalian gastrointestinal microbiota. The other reagents revealed species and matrix-specific differences as well, but overall, except for OMNIgene-GUT, all storage conditions were able to reflect the original microbial composition in chicken, whereas in pig feces, RNALater and freezing showed good performance. Interestingly, RNALater revealed similar properties and comparable storage effects to the homemade buffer NAP for chicken, as previously observed by Menke et al. (2017) in the sheep fecal microbial community, but this was not true for our pig microbiota samples in this study.

It should be noted that this evaluation was performed using different sets of pooled samples (*n* = 5). Therefore, we cannot claim that the microbiota profiles observed here represent those from a larger number of individuals. However, we pooled our samples to ensure that an adequate sample volume was available for multiple comparisons, an approach that has been used for prior gut microbiota studies in human and animal gut microbiota research (Lu et al., 2015; Knudsen et al., 2016; Fidler et al., 2020).

## Conclusion

In this study, we showed how key stages of conducting a microbiota study, including sample storage and DNA extraction, can substantially affect the results, and therefore, their biological interpretation in a matrix-dependent manner. Therefore, using a consistent protocol for sample storage and DNA extraction for all sample types in a single study is recommended. As the effects of these technical steps are potentially large compared with the real biological variability that researchers are seeking to explain, standardization is crucial for accelerating progress in livestock microbiota research. This study provided a framework to assist future animal gut microbiota research projects and standardization efforts.

## Data Availability Statement

The main data supporting the findings of this study are available in its Supplementary Material and in the NCBI BioProject database (accession no. PRJNA665378).

## Ethics Statement

Ethical review and approval was not required for the animal study because different sample types from pig and chicken were obtained from conventional slaughterhouses.

## Author Contributions

MG, GW, and AK designed the project. NG, AK, and GW performed the experiments. MG and GW supervised the development of the experiments and analyzed the results. MG, GW, and VK wrote the manuscript. The authors have read and approved the final manuscript.

## Conflict of Interest

The authors declare that the research was conducted in the absence of any commercial or financial relationships that could be construed as a potential conflict of interest.

## Footnotes

1 https://www.drive5.com/usearch/

## Supplementary Material

Supplementary Figure 1 Impact of extraction method on differential abundances of microbiota. Significant (*q* < 0.05) log-fold changes in the abundances of microbial phyla in samples stored at −80°C or in different storage buffers for chicken cecum **(A)**, chicken feces **(B)**, pig ileum **(C)**, and pig feces samples **(D)**. Positive log-fold change indicates an increase in abundance, while negative log-fold change indicates a reduction in abundance over time compared to the freshly extracted samples group in chicken cecum, chicken feces, pig ileum, and pig feces samples. F: Freshly extracted, M: frozen at −80°C, N: NAP buffer, O: OMNIgene-GUT buffer, R: RNALater, Z: ZYMO DNA/RNA Shield.

Supplementary Data File 1 Supporting information tables including (1) Metadata information on the samples used in the study. (2) Information on the purity of DNA extracted in Phase 1, based on 260/280 index. (3) Information on the purity of DNA extracted in Phase 2, based on 260/280 index. (4) Information on the total cell count per gram of digesta material measured by Flow Cytometry. (5) Details of the OTU that exhibited significantly increased/decreased abundances in the chicken cecum digesta stored in different storage media compared to freshly extracted chicken cecum digesta. (6) Details of the OTU that exhibited significantly increased/decreased abundances in the chicken feces samples stored in different storage media compared to freshly extracted chicken feces. (7) Details of the OTU that exhibited significantly increased/decreased abundances in the pig ileum digesta stored in different storage media compared to freshly extracted pig ileum digesta, and (8) Details of the OTU that exhibited significantly increased/decreased abundances in the pig feces samples stored in different storage media compared to freshly extracted pig feces.

## References

[B1] Alexandratos N. Bruinsma J. (2012). “World agriculture towards 2030/2050: the 2012 revision,” in *ESA Working Paper No. 12-03*. ed. FAO (Rome: FAO).

[B2] Bahl M. I. Bergström A. Licht T. R. (2012). Freezing fecal samples prior to DNA extraction affects the Firmicutes to Bacteroidetes ratio determined by downstream quantitative PCR analysis. *FEMS Microbiol. Lett.* 329 193–197. 10.1111/j.1574-6968.2012.02523.x 22325006

[B3] Burbach K. Seifert J. Pieper D. H. Camarinha-Silva A. (2016). Evaluation of DNA extraction kits and phylogenetic diversity of the porcine gastrointestinal tract based on Illumina sequencing of two hypervariable regions. *MicrobiologyOpen* 5 70–82. 10.1002/mbo3.312 26541370 PMC4767427

[B4] Camacho-Sanchez M. Burraco P. Gomez-Mestre I. Leonard J. A. (2013). Preservation of RNA and DNA from mammal samples under field conditions. *Mol. Ecol. Resour.* 13 663–673. 10.1111/1755-0998.12108 23617785

[B5] Cao W. Ma X. Haney P. Xiong X. Douglas J. Wilborn R., et al. (2019). “Maturation of gut microbiota and dramatic shift of microbial composition during canine puppy development,” in *Phi Zeta Research Day Forum*, ed. The Society of Phi Zeta (Alabama, US: Auburn University).

[B6] Chen Z. Hui P. C. Hui M. Yeoh Y. K. Wong P. Y. Chan M. C. W. (2019). Impact of preservation method and 16S rRNA hypervariable region on gut microbiota profiling. *mSystems* 4:e271-18. 10.1128/mSystems.00271-18 30834331 PMC6392095

[B7] Choo J. M. Leong L. E. X. Rogers G. B. (2015). Sample storage conditions significantly influence faecal microbiome profiles. *Sci. Rep.* 5:16350. 10.1038/srep16350 26572876 PMC4648095

[B8] Chung W. J. Cui Y. Chen C. S. Wei W. H. Chang R. S. Shu W. Y. (2017). Freezing shortens the lifetime of DNA molecules under tension. *J. Biol. Phys.* 43 511–524. 10.1007/s10867-017-9466-3 28887655 PMC5696304

[B9] Costea P. I. Zeller G. Sunagawa S. Pelletier E. Alberti A. Levenez F. (2017). Towards standards for human fecal sample processing in metagenomic studies. *Nat. Biotechnol.* 35 1069–1076. 10.1038/nbt.3960 28967887

[B10] Crespo-Piazuelo D. Estellé J. Revilla M. Criado-Mesas L. Ramayo-Caldas Y. Óvilo C. (2018). Characterization of bacterial microbiota compositions along the intestinal tract in pigs and their interactions and functions. *Sci. Rep.* 8:12727. 10.1038/s41598-018-30932-6 30143657 PMC6109158

[B11] Cuív P. Ó Aguirre De Cárcer D. Jones M. Klaassens E. S. Worthley D. L. Whitehall V. L. J. (2011). The effects from DNA extraction methods on the evaluation of microbial diversity associated with human colonic tissue. *Microb. Ecol.* 61 353–362. 10.1007/s00248-010-9771-x 21153634

[B12] Desneux J. Pourcher A. M. (2014). Comparison of DNA extraction kits and modification of DNA elution procedure for the quantitation of subdominant bacteria from piggery effluents with real-time PCR. *MicrobiologyOpen* 3 437–445. 10.1002/mbo3.178 24838631 PMC4287173

[B13] Deusch S. Tilocca B. Camarinha-Silva A. Seifert J. (2015). News in livestock research — use of Omics -technologies to study the microbiota in the gastrointestinal tract of farm animals. *Comput. Struc. Biotechnol. J.* 13 55–63. 10.1016/j.csbj.2014.12.005 26900430 PMC4720016

[B14] Doukhanine E. Bouevitch A. Brown A. Gage-Lavecchia J. Merino C. Pozza L. (2016). OMNIgene^®^∙GUT stabilizes the microbiome profile at ambient temperature for 60 days and during transport. *DNA Genotek*. Available online at: https://www.dnagenotek.com/us/pdf/PD-WP-00042.pdf

[B15] Ducarmon Q. R. Hornung B. V. H. Geelen A. R. Kuijper E. J. Zwittink R. D. (2020). Toward standards in clinical microbiota studies: comparison of three DNA extraction methods and two bioinformatic pipelines. *mSystems* 5:e547-19. 10.1128/mSystems.00547-19 32047058 PMC7018525

[B16] Edgar R. C. (2013). UPARSE: highly accurate OTU sequences from microbial amplicon reads. *Nat. Methods* 10 996–998. 10.1038/nmeth.2604 23955772

[B17] Edgar R. C. (2017). Accuracy of microbial community diversity estimated by closed- and open-reference OTUs. *PeerJ* 5:e3889. 10.7717/peerj.3889 29018622 PMC5631090

[B18] Ferrand J. Patron K. Legrand-Frossi C. Frippiat J. P. Merlin C. Alauzet C. (2014). Comparison of seven methods for extraction of bacterial DNA from fecal and cecal samples of mice. *J. Microbiol. Methods* 105 180–185. 10.1016/j.mimet.2014.07.029 25093756

[B19] Fidler G. Tolnai E. Stagel A. Remenyik J. Stundl L. Gal F. (2020). Tendentious effects of automated and manual metagenomic DNA purification protocols on broiler gut microbiome taxonomic profiling. *Sci. Rep.* 10:3419. 10.1038/s41598-020-60304-y 32099013 PMC7042355

[B20] Galketi A. Upali Marocco E. (2020). “Overview of global meat market devlopments in 2019,” in *Meat Market Review*, ed. FAO (Rome: FAO).

[B21] Greathouse K. L. Sinha R. Vogtmann E. (2019). DNA extraction for human microbiome studies: the issue of standardization. *Genome Biol.* 20:212. 10.1186/s13059-019-1843-8 31639026 PMC6802309

[B22] Guarino M. Norton T. Berckmans D. Vranken E. Berckmans D. (2017). A blueprint for developing and applying precision livestock farming tools: a key output of the EU-PLF project. *Anim. Front.* 7 12–17. 10.2527/af.2017.0103 32704858

[B23] Gupta S. Mortensen M. S. Schjørring S. Trivedi U. Vestergaard G. Stokholm J. (2019). Amplicon sequencing provides more accurate microbiome information in healthy children compared to culturing. *Commun. Biol.* 2:291. 10.1038/s42003-019-0540-1 31396571 PMC6683184

[B24] Haj-Ahmad Z. Haj-Ahmad L. Kim W.-S. Haj-Ahmad Y. (2015). “Viability of microorganisms in saliva & stool collection and transportation preservatives,” in *American Society for Microbiology*, New Orleans, LA. 10.13140/RG.2.1.4580.6248

[B25] Hallmaier-Wacker L. K. Lueert S. Roos C. Knauf S. (2018). The impact of storage buffer, DNA extraction method, and polymerase on microbial analysis. *Sci. Rep.* 8:6292. 10.1038/s41598-018-24573-y 29674641 PMC5908915

[B26] Haworth S. E. White K. S. Côté S. D. Shafer A. B. A. (2019). Space, time and captivity: quantifying the factors influencing the fecal microbiome of an alpine ungulate. *FEMS Microbiol. Ecol.* 95:fiz095. 10.1093/femsec/fiz095 31210274

[B27] Hill C. J. Brown J. R. M. Lynch D. B. Jeffery I. B. Ryan C. A. Ross R. P. (2016). Effect of room temperature transport vials on DNA quality and phylogenetic composition of faecal microbiota of elderly adults and infants. *Microbiome* 4:19. 10.1186/s40168-016-0164-3 27160322 PMC4862223

[B28] Horng K. R. Ganz H. H. Eisen J. A. Marks S. L. (2018). Effects of preservation method on canine (*Canis lupus* familiaris) fecal microbiota. *PeerJ* 2018:e4827. 10.7717/peerj.4827 29844978 PMC5970549

[B29] Ilett E. E. Jørgensen M. Noguera-Julian M. Daugaard G. Murray D. D. Helleberg M. (2019). Gut microbiome comparability of fresh-frozen versus stabilized-frozen samples from hospitalized patients using 16S rRNA gene and shotgun metagenomic sequencing. *Sci. Rep.* 9:13351. 10.1038/s41598-019-49956-7 31527823 PMC6746779

[B30] Kia E. Wagner Mackenzie B. Middleton D. Lau A. Waite D. W. Lewis G. (2016). Integrity of the human faecal microbiota following long-term sample storage. *PLoS One* 11:e0163666. 10.1371/journal.pone.0163666 27701448 PMC5049846

[B31] Klappenbach J. A. Saxman P. R. Cole J. R. Schmidt T. M. (2001). rrndb: the ribosomal RNA operon copy number database. *Nucleic Acids Res.* 29 181–184. 10.1093/nar/29.1.181 11125085 PMC29826

[B32] Klindworth A. Pruesse E. Schweer T. Peplies J. Quast C. Horn M. (2013). Evaluation of general 16S ribosomal RNA gene PCR primers for classical and next-generation sequencing-based diversity studies. *Nucleic Acids Res.* 41:e1. 10.1093/nar/gks808 22933715 PMC3592464

[B33] Knudsen B. E. Bergmark L. Munk P. Lukjancenko O. Priemé A. Aarestrup F. M. (2016). Impact of sample type and DNA isolation procedure on genomic inference of microbiome composition. *mSystems* 1:e95-16. 10.1128/mSystems.00095-16 27822556 PMC5080404

[B34] Lim M. Y. Hong S. Kim B. M. Ahn Y. Kim H. J. Nam Y. D. (2020). Changes in microbiome and metabolomic profiles of fecal samples stored with stabilizing solution at room temperature: a pilot study. *Sci. Rep.* 10:1789. 10.1038/s41598-020-58719-8 32019987 PMC7000387

[B35] Lim M. Y. Song E. J. Kim S. H. Lee J. Nam Y. D. (2018). Comparison of DNA extraction methods for human gut microbial community profiling. *Syst. Appl. Microbiol.* 41 151–157. 10.1016/j.syapm.2017.11.008 29305057

[B36] Love M. I. Huber W. Anders S. (2014). Moderated estimation of fold change and dispersion for RNA-seq data with DESeq2. *Genome Biol.* 15:550. 10.1186/s13059-014-0550-8 25516281 PMC4302049

[B37] Lu Y. Hugenholtz P. Batstone D. J. (2015). Evaluating DNA extraction methods for community profiling of pig hindgut microbial community. *PLoS One* 10:e0142720. 10.1145/2818302 PMC4641665 26560873

[B38] Marchesi J. R. Adams D. H. Fava F. Hermes G. D. A. Hirschfield G. M. Hold G. (2016). The gut microbiota and host health: a new clinical frontier. *Gut* 65 330–339. 10.1136/gutjnl-2015-309990 26338727 PMC4752653

[B39] McMurdie P. J. Holmes S. (2013). phyloseq: an r package for reproducible interactive analysis and graphics of microbiome census data. *PLoS One* 8:e61217. 10.1371/journal.pone.0061217 23630581 PMC3632530

[B40] Menke S. Gillingham M. A. F. Wilhelm K. Sommer S. (2017). Home-made cost effective preservation buffer is a better alternative to commercial preservation methods for microbiome research. *Front. Microbiol.* 8:102. 10.3389/fmicb.2017.00102 28197142 PMC5281576

[B41] Metzler-Zebeli B. U. Lawlor P. G. Magowan E. Zebeli Q. (2016). Effect of freezing conditions on fecal bacterial composition in pigs. *Animals* 6:18. 10.3390/ani6030018 26927191 PMC4810046

[B42] Oksanen J. Blanchet F. G. Kindt R. Legendre P. Solymos P. Simpson G. L. (2016). *Vegan: Community Ecology Package, R package version 2.3-5.*

[B43] Panek M. Čipčić Paljetak H. Barešić A. Perić M. Matijašić M. Lojkić I. (2018). Methodology challenges in studying human gut microbiota-Effects of collection, storage, DNA extraction and next generation sequencing technologies. *Sci. Rep.* 8:5143. 10.1038/s41598-018-23296-4 29572539 PMC5865204

[B44] Pollock J. Glendinning L. Wisedchanwet T. Watson M. (2018). The madness of microbiome: attempting to find consensus “best practice” for 16S microbiome studies. *Appl. Environ. Microbiol.* 84:e26-17. 10.1128/AEM.02627-17 29427429 PMC5861821

[B45] Rådström P. Knutsson R. Wolffs P. Lövenklev M. Löfström C. (2004). Pre-PCR processing. *Mol. Biotechnol.* 26 133–146. 10.1385/MB:26:2:133 14764939

[B46] Rintala A. Pietilä S. Munukka E. Eerola E. Pursiheimo J. P. Laiho A. (2017). Gut microbiota analysis results are highly dependent on the 16s rRNA gene target region, whereas the impact of DNA extraction is minor. *J. Biomol. Tech.* 28 19–30. 10.7171/jbt.17-2801-003 28260999 PMC5330390

[B47] Sergeant M. J. Constantinidou C. Cogan T. Penn C. W. Pallen M. J. (2012). High-throughput sequencing of 16s rRNA gene amplicons: effects of extraction procedure, primer length and annealing temperature. *PLoS One* 7:e38094. 10.1371/journal.pone.0038094 22666455 PMC3362549

[B48] Silva G. (2019). *Feeding the World in 2050 and Beyond- Part 1: Productivity Challenges.* Available online at: https://www.canr.msu.edu/news/feeding-the-worldin-2050-and-beyond-part-1 (accessed January 15, 2021).

[B49] Song S. J. Amir A. Metcalf J. L. Amato K. R. Xu Z. Z. Humphrey G. (2016). Preservation methods differ in fecal microbiome stability, affecting suitability for field studies. *mSystems* 1:e21-16. 10.1128/mSystems.00021-16 27822526 PMC5069758

[B50] Stanley D. Geier M. S. Chen H. Hughes R. J. Moore R. J. (2015). Comparison of fecal and cecal microbiotas reveals qualitative similarities but quantitative differences. *BMC Microbiol.* 15:51. 10.1186/s12866-015-0388-6 25887695 PMC4403768

[B51] Vandeputte D. Falony G. Vieira-Silva S. Tito R. Y. Joossens M. Raes J. (2016). Stool consistency is strongly associated with gut microbiota richness and composition, enterotypes and bacterial growth rates. *Gut* 65 57–62. 10.1136/gutjnl-2015-309618 26069274 PMC4717365

[B52] Vandeputte D. Kathagen G. D’hoe K. Vieira-Silva S. Valles-Colomer M. Sabino J. (2017). Quantitative microbiome profiling links gut community variation to microbial load. *Nature* 551 507–511. 10.1038/nature24460 29143816

[B53] Videnska P. Smerkova K. Zwinsova B. Popovici V. Micenkova L. Sedlar K. (2019). Stool sampling and DNA isolation kits affect DNA quality and bacterial composition following 16S rRNA gene sequencing using MiSeq Illumina platform. *Sci. Rep.* 9:13837. 10.1038/s41598-019-49520-3 31554833 PMC6761292

[B54] Wesolowska-Andersen A. Bahl M. Carvalho V. Kristiansen K. Sicheritz-Pontén T. Gupta R. (2014). Choice of bacterial DNA extraction method from fecal material influences community structure as evaluated by metagenomic analysis. *Microbiome* 2:19. 10.1186/2049-2618-2-19 24949196 PMC4063427

[B55] Yan W. Sun C. Zheng J. Wen C. Ji C. Zhang D. (2019). Efficacy of fecal sampling as a gut proxy in the study of chicken gut microbiota. *Front. Microbiol.* 10:2126. 10.3389/fmicb.2019.02126 31572332 PMC6753641

[B56] Zheng D. Liwinski T. Elinav E. (2020). Interaction between microbiota and immunity in health and disease. *Cell Res* 30 492–506. 10.1038/s41422-020-0332-7 32433595 PMC7264227

[B57] Zhou X. Nanayakkara S. Gao J.-L. Nguyen K.-A. Adler C. J. (2019). Storage media and not extraction method has the biggest impact on recovery of bacteria from the oral microbiome. *Sci. Rep.* 9:14968. 10.1038/s41598-019-51448-7 31628387 PMC6802381

[B58] Zymo Research. (2021). *Prevent Nucleic acid Degradation Before Extraction.* Available online at: https://www.zymoresearch.com/blogs/blog/prevent-nucleic-acid-degradation (accessed January 15, 2021).

