# Supplementary figures and images for: Toward Best Practice in Livestock Microbiota Research: A Comprehensive Comparison of Sample Storage and DNA Extraction Strategies
Source: Front Microbiol. 2021 Feb 23;12:627539. doi: 10.3389/fmicb.2021.627539 (PMC7940207; doi:10.3389/fmicb.2021.627539)

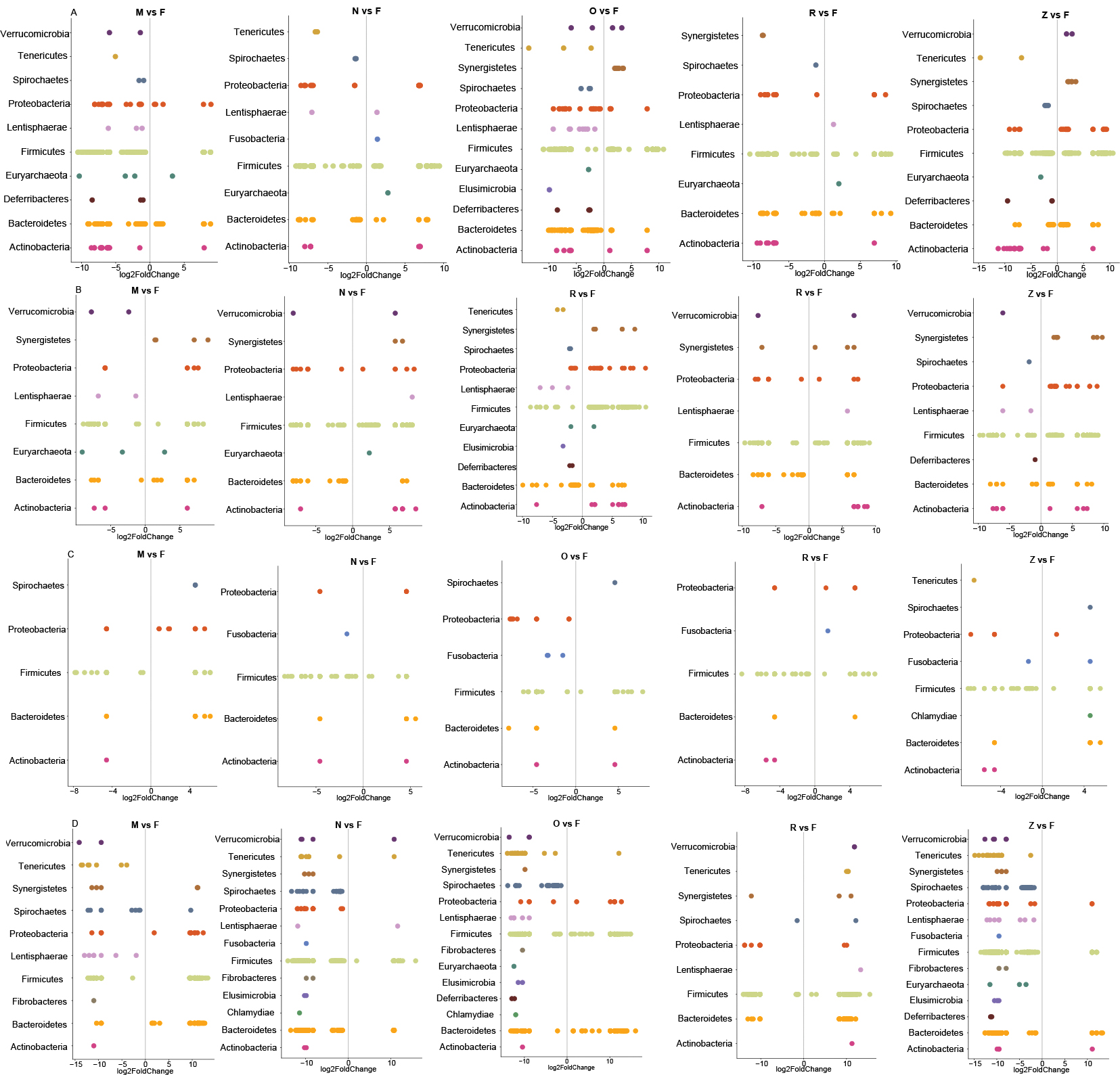

Supplement: Supplementary Figure 1 — Impact of extraction method on differential abundances of microbiota. Significant (q < 0.05) log-fold changes in the abundances of microbial phyla in samples stored at −80°C or in different storage buffers for chicken cecum (A), chicken feces (B), pig ileum (C), and pig feces samples (D). Positive log-fold change indicates an increase in abundance, while negative log-fold change indicates a reduction in abundance over time compared to the freshly extracted samples group in chicken cecum, chicken feces, pig ileum, and pig feces samples. F: Freshly extracted, M: frozen at −80°C, N: NAP buffer, O: OMNIgene-GUT buffer, R: RNALater, Z: ZYMO DNA/RNA Shield. [file Image_1.JPEG]
